# The pharmacokinetic and safety profile of raltegravir and ribavirin, when dosed separately and together, in healthy volunteers

**DOI:** 10.1186/1758-2652-13-S4-O33

**Published:** 2010-11-08

**Authors:** J Ashby, LJ Garvey, OW Erlwein, H Lamba, R Weston, K Legg, MO McClure, L Dickinson, A D'Avolio, DJ Back, A Winston

**Affiliations:** 1 Imperial College Healthcare NHS Trust, GU/HIV Medicine, London, UK; 2 Imperial College London, Section of Infectious Diseases, HIV Medicine, London, UK; 3 Imperial College London, Section of Infectious Diseases, London, UK; 4 University of Liverpool, Department of Pharmacology and Therapeutics, Liverpool, UK; 5 University of Torino, Pharmacokinetics and Pharmacogenetics Laboratory, Torino, Italy

## Purpose of the study

Treatment of chronic hepatitis C virus (HCV) infection in HIV-1 co-infected individuals remains challenging due to numerous factors including drug-drug interactions. The aim of this study was to assess the safety and pharmacokinetic (PK) profile of raltegravir, a recently licensed antiretroviral agent, and ribavirin, when dosed separately and together.

## Methods

Fourteen healthy volunteers (mean (standard deviation) age 35 (10) years, 71% male) entered this phase I PK study and received single dose ribavirin (800 mg) on day 1 (*phase 1*). Following a wash-out period, subjects received raltegravir (400 mg twice daily) on days 15-19 (*phase 2*) and single dose ribavirin (800 mg) with raltegravir (400 mg) on day 20 (*phase 3*). Intensive PK sampling was undertaken on days 1, 19 and 20 and differences in geometric mean ratios (GMR) for PK parameters between study periods assessed.

## Results

No statistically significant differences in PK parameters were observed for raltegravir between *phases 2* versus *3*. A statistically significant decrease in maximum plasma concentration (Cmax) and increase in time to maximum plasma concentration (Tmax) was observed for ribavirin in *phase 3* compared to *phase 1* (GMR (95% CI) 0.79 (0.62 - 1.00) and 1.39 (1.08 - 1.78), respectively; Table 1) whereas no significant differences in other ribavirin PK parameters were observed between study phases including area under-time-curve (AUC) or minimum observed plasma concentration (Cmin). No clinically significant safety concerns were reported.

**Table 1:** 

	mean(95% CI)	mean(95% CI)	GMR (95% CI)
Ribavirin PK parameters	phase I (ribavirin alone)	phase 3 (ribavirin with raltegravir)	

T ½, h	6.04 (5.29 - 6.90)	6.77 (5.56 - 8.25)	1.12 (0.86 - 1.46)

Tmax, h	1.61 (1.12 - 2.11)	2.23 (1.65 - 3.01)	1.39 (1.08 - 1.78)

Cmax, ng/mL	630.09 ( 490.91 - 808.54)	496.71 (407.38 - 605.76)	0.79 (0.62 - 1.00)

Cmin, ng/mL	184.71 (148.59 - 229.61)	186.98 (157.83 - 221.56)	1.01 (0.87 - 1.18)

AUC0-12	3325.83 (2703.34 - 4091.66)	2941.03 (2323.27 - 3722.20)	0.88 (0.73 - 1.07)

## Conclusions

The PK profile of ribavirin is altered when administered with raltegravir (reduced Cmax and increased Tmax). This is unlikely to be of clinical significance or have an impact on the antiviral effects of ribavirin in HIV-1 and HCV co-infected subjects.

